# Purified Inactivated Zika Vaccine Candidates Afford Protection against Lethal Challenge in Mice

**DOI:** 10.1038/s41598-018-34735-7

**Published:** 2018-11-07

**Authors:** Whitney R. Baldwin, Jill A. Livengood, Holli A. Giebler, Janae L. Stovall, Karen L. Boroughs, Stephanie Sonnberg, Kelly J. Bohning, Elizabeth A. Dietrich, Yee Tsuey Ong, Hoang K. Danh, Hetal K. Patel, Claire Y.-H. Huang, Hansi J. Dean

**Affiliations:** 1 Takeda Vaccines Inc, Cambridge, MA USA; 2 Arboviral Diseases Branch, Division of Vector-Borne Diseases, Centers for Disease Control and Prevention, Fort Collins, Colorado, USA

## Abstract

In response to the 2016 global public health emergency of international concern announced by the World Health Organization surrounding Zika virus (ZIKV) outbreaks, we developed a purified inactivated Zika virus vaccine (PIZV) candidate from ZIKV strain PRVABC59, isolated during the outbreak in 2015. The virus isolate was plaque purified, creating six sub-isolated virus stocks, two of which were selected to generate PIZV candidates for preclinical immunogenicity and efficacy evaluation in mice. The alum-adjuvanted PIZV candidates were highly immunogenic in both CD-1 and AG129 mice after a 2-dose immunization. Further, AG129 mice receiving 2 doses of PIZV formulated with alum were fully protected against lethal ZIKV challenge and mouse immune sera elicited by the PIZV candidates were capable of neutralizing ZIKVs of both African and Asian genetic lineages *in vitro*. Additionally, passive immunization of naïve mice with ZIKV-immune serum showed strong positive correlation between neutralizing ZIKV antibody (NAb) titers and protection against lethal challenge. This study supported advancement of the PIZV candidate toward clinical development.

## Introduction

Zika virus (ZIKV) is a member of *Flaviviridae*, which includes other human pathogenic flaviviruses such as West Nile virus (WNV), Japanese encephalitis virus (JEV), Yellow Fever virus (YFV), dengue virus (DENV) and tick-borne encephalitis virus (TBEV). Flaviviruses possess a single-stranded, positive sense RNA genome, approximately 11 Kb long. One open reading frame encodes a single polyprotein that is processed by host and viral proteases to produce three structural proteins, capsid (C), premembrane/membrane (prM/M), and envelope (E), and seven non-structural proteins, NS1, NS2A, NS2B, NS3, NS4A, NS4B and NS5^1^. The prM/M and E proteins form the outer virion envelope in an icosahedral arrangement of 90 E protein dimers. The E protein, involved in viral attachment and fusion with the host cell membrane, is the main target of host neutralizing antibodies (NAbs) against viral infection^2^.

ZIKV was first identified and isolated from a sentinel monkey in a Ugandan forest in 1947^3,4^, and emerged as a global health threat in December 2015^5^. Prior to 2007, only a few human cases of ZIKV infection had been reported in Africa and Southeast Asia, with relatively unremarkable clinical consequences^6–9^. However, ZIKV has been linked to severe fetal abnormalities and neurological diseases in both newborns and adults during the recent epidemic in the Americas^10–13^ and retrospectively in French Polynesia^14–16^. This global spread of ZIKV in previously naïve populations is reminiscent of other emerging arboviral infectious diseases, such as chikungunya. As of July 2018, more than 100 countries and territories had reported cases of ZIKV transmission^17^. Whereas the initial explosive outbreak has waned in some countries, ZIKV has continued to spread geographically^18^. While ZIKV transmission is primarily mosquito-borne, it can also be transmitted sexually and vertically, causing perinatal and congenital ZIKV infections^12,19^. The devastating impact of Zika virus infection on a developing fetus underlies the global need for the rapid development of a vaccine as part of comprehensive disease prevention measures including education and vector control. Purified inactivated vaccines have been developed and safely utilized for the prevention of diseases caused by other flaviviruses, including JEV and TBEV^20^. Herein, we describe the derivation and initial pre-clinical development of an inactivated Zika vaccine, the results of which support further pre-clinical and clinical development of PIZV.

## Results

### Derivation and characterization of ZIKV sub-isolates

We chose ZIKV strain PRVABC59 for PIZV development based on the criteria that it was isolated from a serum sample from a hospitalized patient who was infected in Puerto Rico in late 2015 during the most recent ZIKV outbreak in the Americas, and the isolation history of the virus is well documented. To remove any potential adventitious agents that may have been present in the virus isolate (from the human specimen and/or during initial culture), and to generate a virus stock adapted for efficient growth in Vero cells for virus production during manufacturing, we further plaque-purified the virus isolate to derive 6 sub-isolate seed stocks. We first amplified the virus in Vero cells in serum-free medium to make our passage 1 stock (P1), and then conducted three rounds of virus plaque purification (P2-4) (selecting the largest and clearest plaques) to obtain 6 sub-isolates (a-f). These sub-isolates were amplified twice more in Vero cells to generate high titer passage 6 stocks (P6) (Table 1).

**Table 1:** Generation and characterization of ZIKV sub-isolates used to derive PIZV candidates.

	Virus stock generation	Characterization
CDC	Virus isolate amplified in Vero (2×) and C6/36 (1×)(V2/C6-1)	Plaque titer/phenotype, identity by RT-PCR, mycoplasma and togavirus testing
P1	Virus amplification in Vero	TCID_50_ titer
P2	Plaque purification of P1	plaque phenotype
P3	Plaque purification of P2	plaque phenotype
P4	Plaque purification of P3	plaque phenotype
P5	Amplification of P4 plaques (a-f sub-isolates) in Vero	TCID_50_ titer
P6	Amplification of P5 virus (a-f sub-isolates) in Vero	TCID_50_ titer, plaque phenotype, full genome sequencing, growth kinetics in Vero
P7	Amplification of P6 (b and e sub-isolates) in Vero; Used for deriving PIZV	Relative purity (SDS-PAGE), antigenicity (immunoblot, ELISA), EM, completeness of inactivation (serial cell culture passaging)

**Figure 1:**
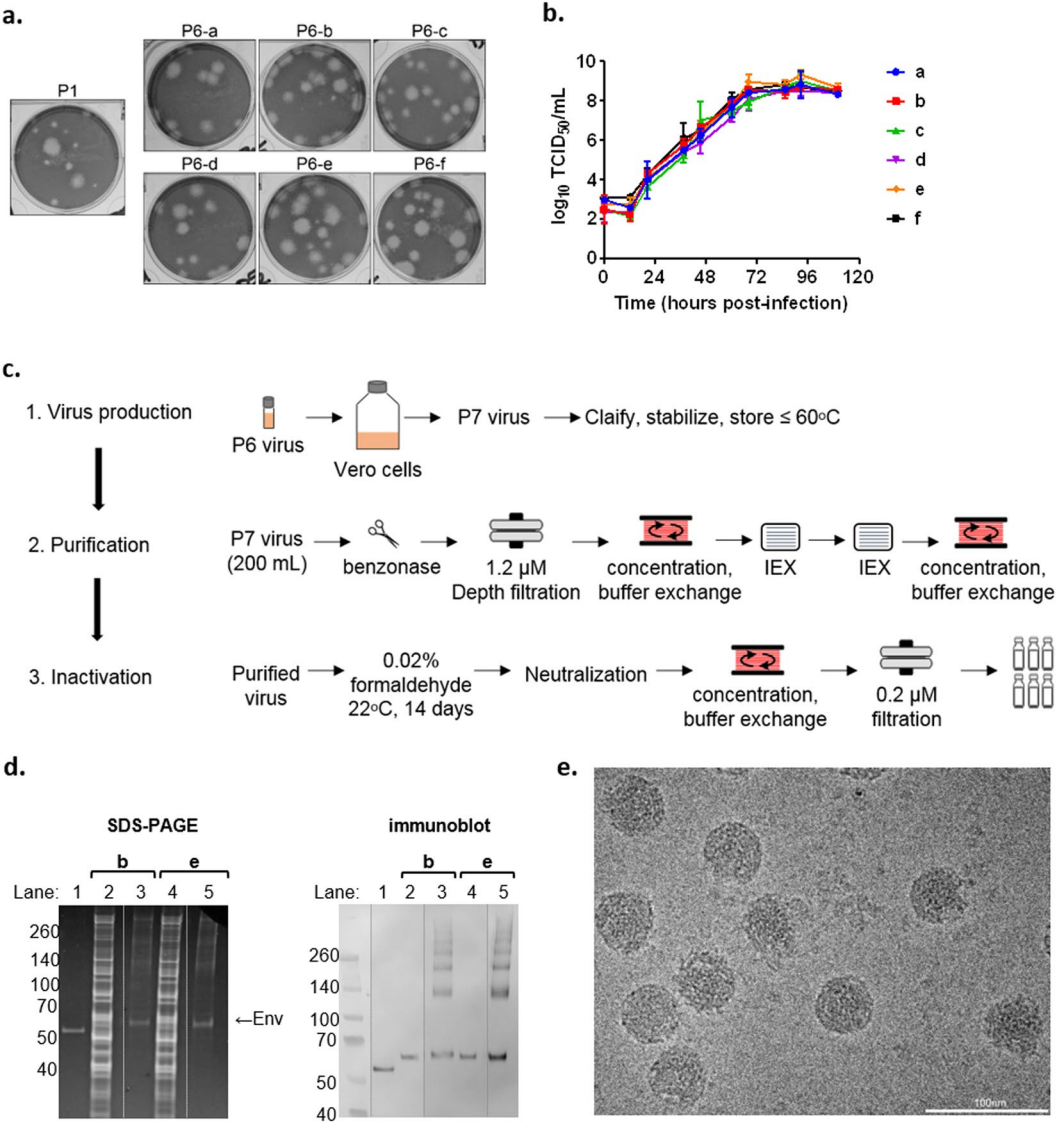
Characterization and preparation of PIZV candidates. (**a**) Plaque phenotype of ZIKV PRVABC59 P6 sub-isolates, compared to ZIKV PRVABC59 P1 stock. (**b**) Growth kinetics of ZIKV PRVABC59 P6 sub-isolates. (**c**) Flowchart of PIZV preparation. (**d**) Left panel: total protein on SDS-PAGE. Right panel: Immunoblot analysis detecting ZIKV E. Lane 1: recombinant ZIKV sE; lane 2 and 4: P7 virus supernatant; lane 3 and 5: PIZV made from P7 virus. (**e**) Representative electron micrograph of PIZV-e at 110,000X magnification.

Phenotypic analysis of the P6 sub-isolate stocks revealed that after plaque purification, each stock consisted of a higher population of large plaques compared with the P1 virus which had a higher proportion of small plaques (Fig. 1a). Growth kinetics analysis of the P6 sub-isolate stocks in Vero cells cultured in serum-free medium showed that all replicated efficiently and reached a peak titer of 8–9 log_10_ TCID_50_/mL between 3 and 4 days post infection (pi) (Fig. 1b).

Since the E protein is the major antigen of an inactivated flavivirus vaccine, we sequenced the E gene of the P6 sub-isolate stocks to ensure selection of optimal candidates without any E mutations that might alter NAb epitopes (Table 2). The P6-a, P6-c, P6-d and P6-f virus stocks contained a G → T mutation at nucleotide 1965 (G1965T), resulting in an amino acid substitution of Val → Leu at E protein residue 330 (E-V330L), whereas P6-b and P6-e stocks were identical relative to the PRVABC59 reference sequence, which was derived through deep sequencing of the human specimen prior to virus isolation (GenBank access: KU501215.1)^21^. Interestingly, we found that both E-330 V and L virus variants co-presented in a Vero-2 virus stock (Vero-1 represents the initial virus isolation stock). These results suggest that the E-V330L mutation was likely one of the major Vero cell-adaptive mutations, evolving early during virus isolation in Vero cells.

**Table 2:** Genetic characterization of ZIKV P6 (a-f) sub-isolates.

clone	Mutations identified in E		Additional mutations identified in genome	
	genome nt position	amino acid	genome nt position	amino acid
P6-a	G1965T	E-V330L	nd	nd
P6-b	T1405G	(silent)	T2781G	NS1-W98G
P6-c	G1965T	E-V330L	nd	nd
P6-d	G1965T	E-V330L	nd	nd
P6-e	none	None	T2781G	NS1-W98G
P6-f	G1965T	E-V330L	nd	nd

The sequence initially determined for ZIKV strain PRVABC59 was used as a reference sequence (GenBank access: KU501215.1). nd indicates not determined.

Based upon our initial criteria, PRVABC59 P6-b and P6-e (containing no E mutations) were selected and subjected to full genome sequencing to identify other potential cell-adaptive mutations. Sequence analysis revealed both sub-isolates contained a single T2781G substitution, resulting in a NS1-W98G substitution. The NS1-98 Trp is highly conserved among different strains of ZIKV, and is located in the intertwined loop of the NS1 wing domain, which has been implicated in membrane association, interaction with envelope protein and potentially hexameric NS1 formation^22–25^. However, the NS1-Trp98Gly substitution did not negatively impact viral replication efficiency in Vero cells. Therefore, the P6-b and P6-e sub-isolates were selected for development of PIZV candidates (Table 1).

### Preparation and characterization of PIZV

To generate research grade PIZV material for animal studies, P6-b and P6-e sub-isolate stocks were cultured in Vero cells using serum-free medium to produce passage 7 (P7) stocks for virus purification and inactivation (Table 1). ZIKV P7 stocks were purified using depth filtration and ion-exchange chromatography and inactivated with formalin, conditions of which were empirically determined (Fig. 1c). PIZV material used in this study was inactivated for 14 days, which was double the duration required for complete virus inactivation in a preliminary study of process development. To confirm completeness of inactivation of the purified and inactivated virus, 2 sequential Vero cell culture amplifications of the inactivated material were performed. We determined that the purified material (initial virus stocks at 8.6–8.9 log_10_ TCID_50_/mL) could be effectively inactivated with 0.02% formaldehyde treatment for 14 days at room temperature with frequent mixing. Overall, the small laboratory scale production of P7 virus (200 mL of 8.6–8.9 log_10_ TCID_50_/mL) yielded a sufficient amount of PIZV for proof of concept studies (0.35–0.45 mg).

The relative purity of PIZV candidates was assessed by total protein staining after separation by SDS-PAGE (Fig. 1d). Extensive purification was reflected by a substantial increase in the ratio of E protein to host cell protein contaminants following purification, inactivation and concentration of PIZV material. Additionally, the laddered protein bands shown in the PIZV samples were recognized by anti- ZIKV E antibody in the Western blot with sizes equivalent to multimeric E proteins (Fig. 1d), suggesting the bands likely resulted from covalent cross-linking of the E protein after formalin treatment (Fig. 1d).

As an assurance that the inactivation process did not significantly alter the viral antigenic property, a sandwich ELISA utilizing several monoclonal antibodies (mAbs 4G2, EDE C8, EDE C10 and 1176–56) was used to measure binding epitopes on the PIZV-e candidate (Supplementary Fig. S1). The E dimer epitope (EDE) mAbs, EDE1 C8 and EDE1 C10, originally isolated from DENV-infected patients, bind across the E dimer interface (quaternary epitopes on virion surface)^26,27^ and have high ZIKV cross-neutralizing and cross-protective capacity^28–30^. These mAbs can also bind to recombinant soluble E (sE) monomers, in which binding results in the formation of E dimers^27^. Utilizing EDE mAbs (as capture antibodies) and a 4G2 mAb (flavivirus cross-reactive for antigen detection), the ELISA demonstrated that similar amounts of PIZV input and sE resulted in a comparable optical density as 5.8 log_10_ TCID_50_ of live ZIKV input (Supplementary Fig. S1). In contrast, the mouse mAb 1176–56, which was raised against recombinant ZIKV sE, did not bind to either live ZIKV or the PIZV material. Additionally, electron microscopic analysis revealed that the PIZV material contained many intact, 44–50 nm round particles with a textured appearance, dense interiors and either smooth or uneven borders, consistent with the expected appearance of ZIKV (Fig. 1e). Interestingly, the majority of the particles (about 85%) had a smooth surface morphology, suggesting a high proportion of PIZV consisted of mature viral particles. Overall, these results indicated that the PIZV preparation retains mostly whole viral particles and displays antigenic epitopes recognized by two highly neutralizing and flavivirus cross-protective human mAbs. Concentration of purified inactivated ZIKV antigen was determined by Bradford assay prior to formulation into drug product in PBS (with and without Alum) (PIZV).

### Immunogenicity of PIZV candidates in immunocompetent CD1 mice

**Figure 2:**
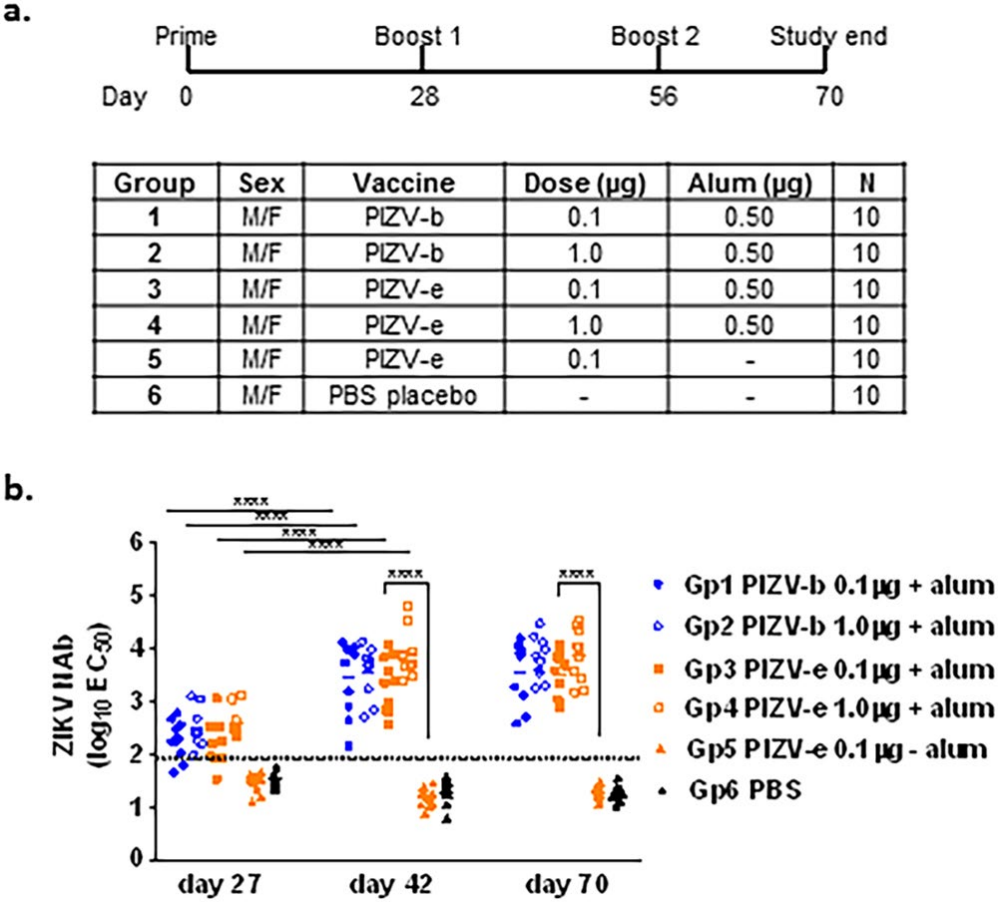
PIZV immunogenicity in CD1 mice. (**a**) Test groups and schedule of dosing. (**b**) Serum NAb titers of immunized CD-1 mice as determined by RVP neutralization assay. Solid lines represent the geometric mean titer (GMT) of EC_50_ for each group. The limit of detection (1.9 log_10_) is represented by a dashed line. Samples with a titer below the detection limit were included in the GMT calculation. Data were analyzed by one-way ANOVA with Tukey’s multiple comparisons test: **** indicates p < 0.0001.

We assessed the immunogenicity of our research grade PIZV-b and -e candidates in 6-week old male and female CD-1 mice (Fig. 2a). NAbs were detected in 34/40 (85%) of mice 27 days after receiving the first dose of either PIZV candidate containing alum (groups 1–4), compared with 0/10 (0%) of mice in the non-adjuvanted vaccine group (group 5) and 0/10 (0%) in the PBS placebo control group (group 6) (Fig. 2b). The NAb titers increased ~10 fold following a second dose, reaching geometric mean titers (GMTs) of 3.4, 3.5, 3.3, and 3.9 log_10_ for groups 1–4 respectively, but were not boosted after a third dose. There was no significant difference in the NAb GMTs observed between groups vaccinated with the same dose of PIZV-b or PIZV-e (group 1 vs group 3 and group 2 vs group 4) at any time point tested. Additionally, no significant difference in NAb GMT was observed between groups of mice vaccinated with a low or high dose of PIZV candidates (groups 1 and 3 vs groups 2 and 4). Finally, groups of mice receiving a low dose of non-adjuvanted vaccine did not develop measurable NAb titers, despite receiving three doses (group 5).

### Immunogenicity and efficacy of PIZV candidates in interferon receptor-deficient AG129 mice

**Figure 3:**
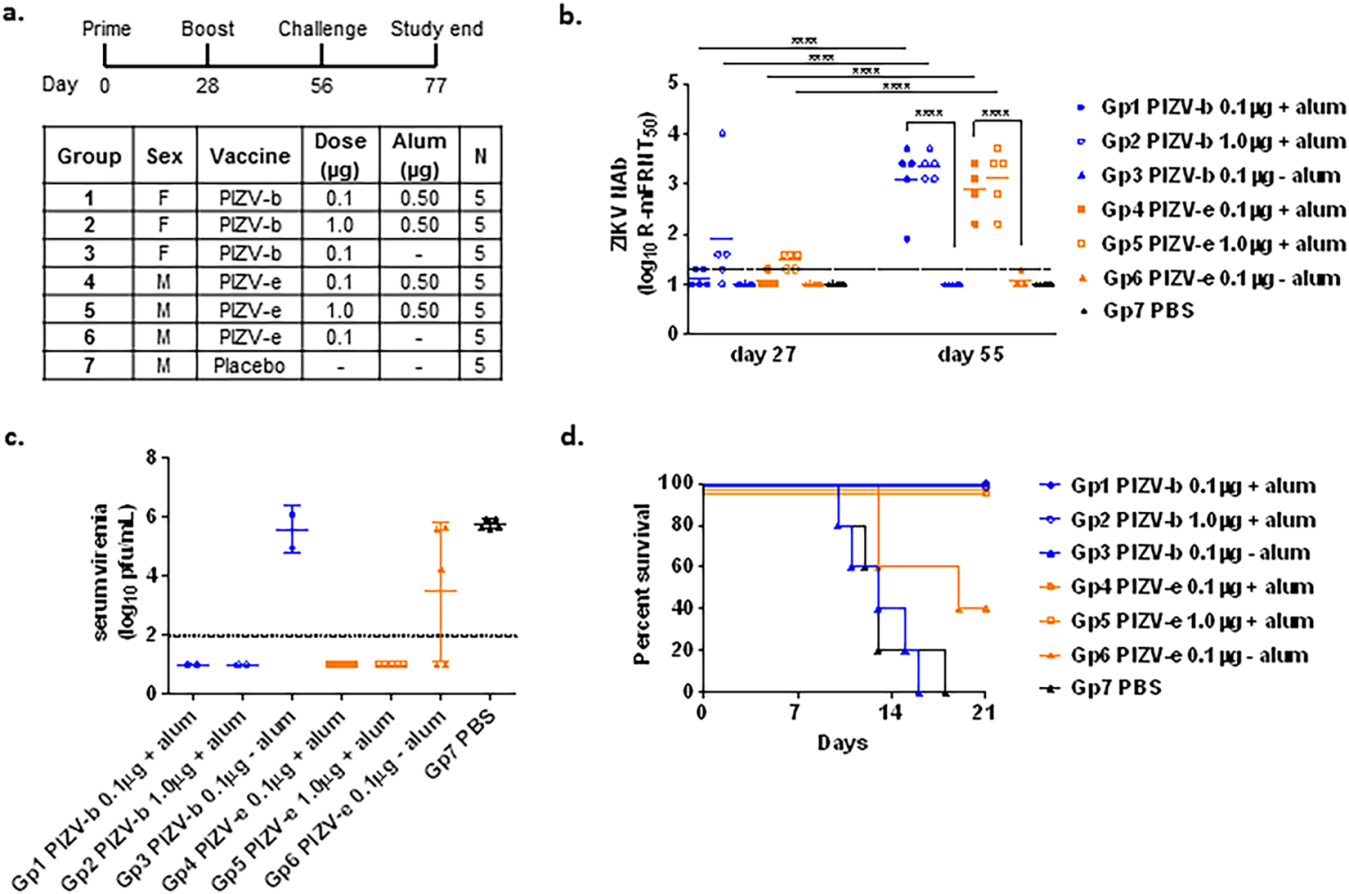
PIZV immunogenicity and efficacy in AG129 mice. (**a**) Test groups and schedule of dosing. (**b**) Serum NAb titers as determined by R-mFRNT of immunized AG129 mice. Solid lines represent the group GMT of the reciprocal serum dilution required for at least 50% reduction of viral foci. The limit of detection (1.3 log_10_) is represented by a dashed line. Animals with no detectable titer (<1.3) were assigned a titer of 1.0 for visualization and GMT calculation. Data were analyzed by one-way ANOVA with Tukey’s multiple comparisons test: ****indicates p < 0.0001. (**c**) Infectious virus titers of individual mouse serum samples two days post-challenge, shown as pfu/mL. Solid lines represent the GMT of a group. Error bars represent standard deviation. The limit of detection (2.0 log_10_) is represented by a dashed line. Animals with no detectable titer (<2.0) were assigned a titer of 1.0 for visualization and GMT calculation. (**d**) Kaplan-Meier survival curve of test groups post-challenge.

The immunogenicity and protective efficacy of the research grade PIZV candidates was assessed in 4- week old male and female AG129 mice (Fig. 3a). Only groups 2 and 5, which received the high dose (1.0 µg) of alum-adjuvanted vaccine, had a detectable (≥1.3 log_10_) NAb GMT after a single dose (Fig. 3b). In contrast, all groups of mice receiving either the low (0.1 µg) or high dose (1.0 µg) of PIZV with alum (groups 1, 2, 4 and 5) developed high NAb titers (GMTs of 2.9–3.3 log_10_) after a second dose. After receiving two doses of vaccine, there was no statistical difference in group GMTs between groups of mice receiving alum-adjuvanted vaccine, regardless of the dosage or the derivation from the P7 stock. With the exception of 1 animal, groups of mice vaccinated with non-alum adjuvanted vaccine (groups 3 and 6) failed to elicit detectable NAbs, even after a second dose. Finally, serum collected on day 55 was pooled according to test group and assessed for neutralizing activity against African (MR766 and DakAr41524) and Asian (PRVABC59, P6-740 andFSS13025) ZIKV strains using a plaque reduction neutralization test (PRNT). As demonstrated in Table 3, serum collected from alum-adjuvant vaccinated mice neutralized all ZIKV strains to similar extents (between 2.5–3.1 log_10_).

**Table 3:** Reciprocal log_10_ PRNT_50_ titers of pooled sera from PIZV vaccinated AG129 mice against various ZIKV strains. The limit of detection of the assay was 1.3 log_10_.

Group	ZIKV NAb (log_10_ PRNT_50_)				
	*MR766*	*DakAr41524*	*P6-740*	*FSS13025*	*PRVABC59*
1	2.5	2.8	nd*	2.8	2.8
2	2.8	3.1	3.1	3.1	2.8
3	<1.3	<1.3	<1.3	<1.3	<1.3
4	2.5	2.5	2.5	2.8	2.5
5	2.8	2.8	2.8	3.1	2.8
6	<1.3	<1.3	<1.3	<1.3	<1.3
7	<1.3	<1.3	<1.3	<1.3	<1.3

^*^nd: not determined .

Following the 2-dose (1 prime dose and 1 boost dose) immunization schedule, vaccinated and control AG129 mice were challenged at day 56 with 10^4^ pfu of ZIKV PRVABC59, which is more than 100-fold higher than the 50%-lethal dose (LD_50_) of the virus (see methods for details). Groups 1, 2, 4 and 5 vaccinated with a low or high dose of either PIZV candidate formulated with alum were fully protected from lethal ZIKV challenge without any weight loss or clinical sign of illness (Supplementary Fig. S2). In addition, no viremia was detected in these mice two days post ZIKV challenge (Fig. 3c, LOD = 2.0 log_10_ pfu/mL). In contrast, challenge of all sham-vaccinated mice resulted in high viremia on day 2 post challenge (5.6–5.9 log_10_ pfu/mL) and morbidity/mortality between days 10 and 18 post challenge (median survival time = 13 days) (Fig. 3c,d). Mice vaccinated with non-adjuvanted vaccine also exhibited viremia (5.0–6.1 log_10_ pfu/mL) and succumbed to illness between days 10 and 19 (median survival time = 13 and 19 days, group 3 and 6, respectively) (Fig. 3c, d).

### Protection of AG129 mice through passive transfer of anti-ZIKV immune sera

**Figure 4:**
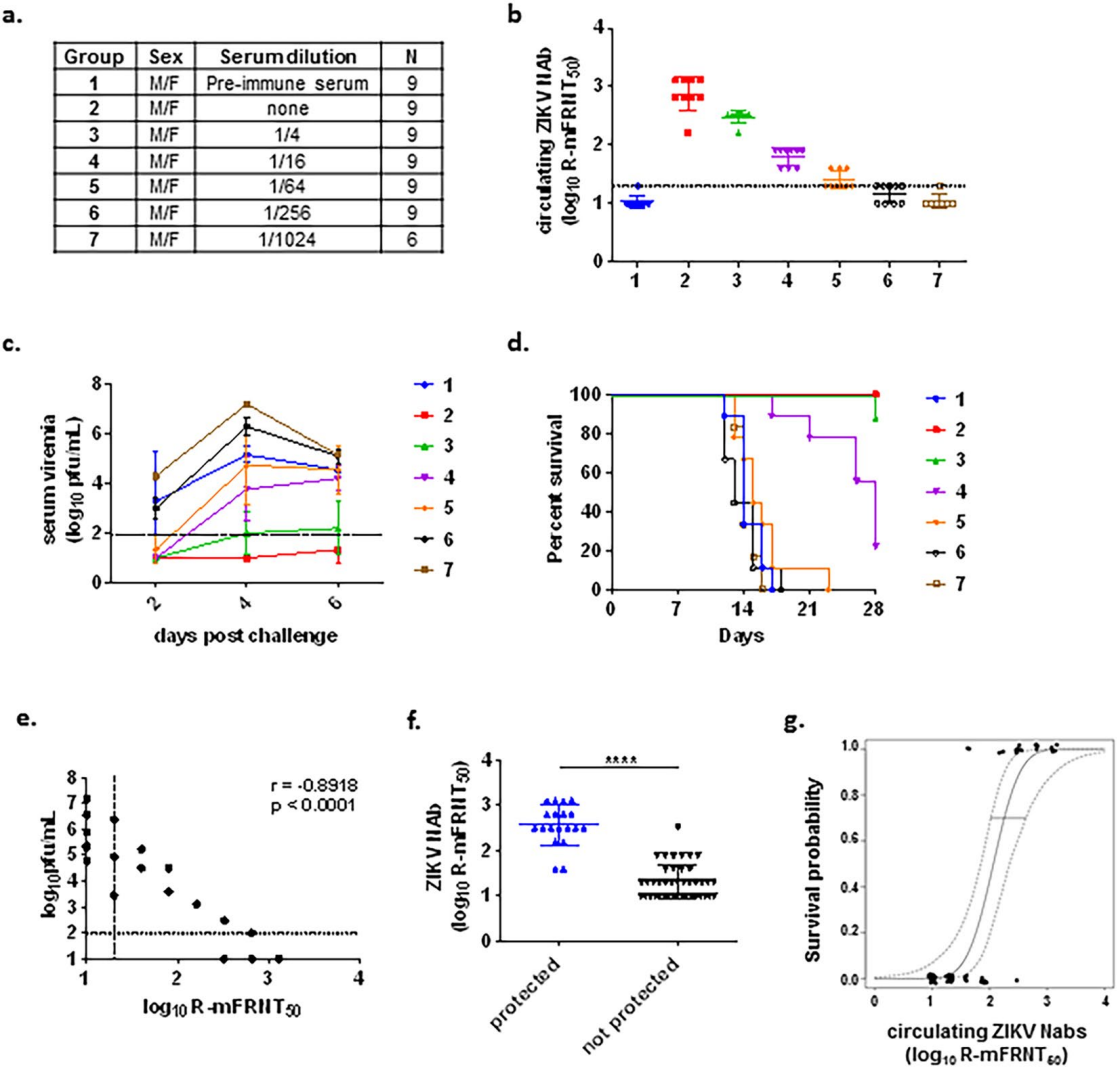
Passive immunization of ZIKV immune sera in AG129 mice. (**a**) Test groups. (**b**) Circulating serum NAb levels measured by R-mFRNT, prior to challenge. Solid lines with error bars represent the GMT with standard deviation. The limit of detection (1.3 log_10_) is represented by a dashed line. Animals with no detectable titer (<1.3) were assigned a titer of 1.0 for visualization and GMT calculation. (**c**) Infectious viremia titers of individual mice post-challenge. Solid lines with error bars represent the GMT with standard deviation. The limit of detection (2.0 log_10_) is represented by a dashed line. Animals with no detectable titer (<2.0) were assigned a titer of 1.0 for visualization and GMT calculation. (**d**) Kaplan-Meier survival curve of test groups post-challenge. (**e**) Correlation analysis of circulating serum NAb titers at the time of challenge and viremia after challenge. Data used for this analysis were from 3 mice/group based on the group peak viremia day. Horizontal dashed line represents the limit of detection of the plaque assay, and vertical dashed line represents the limit of detection of the R-mFRNT assay. A Spearman rank correlation test was used to determine a rho and p value. (**f**) Comparison of circulating serum NAb titers following passive transfer in protected and unprotected test groups at 28 days post challenge. Geometric mean is indicated by a solid bar. The **** symbol indicates a p value < 0.0001 using a Wilcoxon rank test. (**g**) Fitted logistic regression model for survival as a function of circulating NAb titers following passive transfer. An inverse prediction of 70% is indicated by a horizontal line. Dashed lines indicate a 95% confidence interval.

We sought to identify and demonstrate a protective level of anti-ZIKV NAb in the highly-ZIKV-susceptible AG129 mouse model. Serum collected post-challenge from surviving immunized AG129 mice was pooled, serially diluted in PBS and transferred into 14-week old AG129 mice by intraperitoneal (i.p.) injection. Pre-immune AG129 mouse serum was used as placebo control (group 1) (Fig. 4a). Circulating NAb titers from sera collected following passive transfer and prior to ZIKV challenge were determined (GMT of <1.3, 2.9, 2.5, 1.8, 1.4, <1.3, and <1.3 log_10_ for groups 1–7, respectively) (Fig. 4b). Serum samples collected two, four and six days post challenge were analyzed for infectious viremia (N = 2–3/group/day) (Fig. 4c). Signs of disease began appearing 12 days after challenge in the sham-treated group (group 1) and groups receiving the highest 2 dilutions of ZIKV immune sera (groups 6 and 7), with a corresponding loss in weight (Supplemental Fig. S2). Median survival time of the sham group was 14 days. Mice receiving undiluted ZIKV immune sera (group 2) were 100% (9/9) protected from illness and weight loss, while mice receiving 4-fold and 16-fold diluted sera (groups 3 and 4) were partially protected (89% and 22% survival, respectively) (Fig. 4d). Only 1 of 9 mice became morbid on day 28 post challenge in group 3, while 7 out of 9 mice in group 4 became ill with a median survival time of 28 days. These results attest to the importance of circulating ZIKV NAb in eliminating disease or prolonging survival from lethal ZIKV challenge. Indeed, correlation analysis using the highest viremia data collected for each group (day 4 or 6 post challenge as shown in Fig. 4c) and the corresponding NAb titers at the time of challenge revealed an inverse correlation between ZIKV NAb titers and peak viremia titers after challenge (r = −0.8918, p < 0.0001) (Fig. 4e). Additionally, NAb titers were significantly higher (p < 0.0001) in animals protected from morbidity (2.6 ± 0.5) than non-protected animals (1.3 ± 0.4) (Fig. 4f). Logistic regression analysis showed that a ZIKV NAb titer of 2.2 log_10_ (95% CI: 2.0–2.7) by a live reporter virus-based micro-foci reduction neutralization assay (R-mFRNT_50_) is predicted to provide a 70% probability of survival (Fig. 4g).

## Discussion

Several ZIKV vaccine platforms are in development, including DNA, mRNA, peptide, recombinant viral vector, live attenuated, and inactivated vaccines^31–37^. While studies have shown that several of these platforms are immunogenic, the safety profile and track record of effectiveness of purified, inactivated flavivirus vaccines makes this an attractive platform for rapid vaccine development and deployment, as described in a ZIKV vaccine target product profile published by the WHO and UNICEF, which outlines the preferred and minimal product characteristics of a vaccine in emergency situations such as an outbreak response^38^. To address this public health need, we developed a purified, inactivated whole virus Zika vaccine candidate derived from PRVABC59, an American outbreak strain of Asian genotype.

The process of plaque purification and adaptation to a serum-free Vero cell vaccine production process allowed us to obtain and select virus stocks with efficient and reproducible growth characteristics to be used for vaccine process scale-up and manufacturing. Sequencing demonstrated that adaptation to Vero cells had no impact on the E protein amino acid composition and introduced a single point mutation in NS1. A research scale production of PIZV generated fully inactivated, whole virion vaccine antigen particles with smooth morphology, which retained multiple ZIKV E antigenic properties, including key neutralization epitopes.

We evaluated the immunogenicity of the PIZV candidates in immunocompetent, outbred CD-1 mice and interferon (IFN) receptor-knockout AG129 mice as well as protective efficacy in AG129 mice. Like other immunocompetent mouse models, CD-1 mice are useful for evaluating the immunogenicity of the PIZV vaccine, but are not susceptible to ZIKV infection through peripheral injection, which best simulates virus transmission through mosquito bite. Due to the lack of both IFN-α/β and IFN-ɣ receptor genes, AG129 mice are highly susceptible to many flaviviruses, such as DENV and ZIKV, even by peripheral inoculation^39–42^. Although immunocompromised, AG129 mice appear to have a largely normal humoral immune response and have been widely used in challenge studies to investigate viral pathogenesis and vaccine efficacy for ZIKV^35,43,44^. Using these two animal models, we investigated the effects of immunization schedule, dose range, and alum-adjuvant on the NAb response. Additionally, the AG129 model was used to test vaccine efficacy in a challenge study using a highly lethal dose of ZIKV. Our data demonstrated that two doses of either 0.1 µg or 1.0 µg of the PIZV candidates, in the presence of alum, elicited robust immune responses in both mouse models and fully protected AG129 mice from morbidity and mortality. Additionally, a NAb response was not detected in mice given PIZV in the absence of alum in either mouse model, supporting inclusion of alum in the PIZV formulation.

The primary objective of this study was to prove the principle that a clonally-derived PIZV vaccine could generate an immune response that correlated with protection. Immunogenicity and efficacy data from pre-clinical animal studies of this PIZV are largely in agreement with pre-clinical data reported from other inactivated, whole-virion vaccine candidates^31,35^. However, it is difficult to make direct comparisons given the different animal models, vaccine doses utilized, neutralizing antibody assay platforms, ZIKV challenge strains, and challenge routes used in these studies. For example, LaRocca *et al*. 2016, demonstrated that a single, 1.0 µg dose of alum-adjuvanted inactivated vaccine derived from the PRVABC59 strain provided a NAb response with a PRNT_50_ titer of 15, which protected against viremia following intravenous administration of a Brazilian ZIKV strain in Balb-C mice^31^. Sumathy *et al*. 2017, showed that two 10 μg doses of an alum-adjuvanted inactivated vaccine derived from the East African genotype MR766 strain provided full protection against mortality following lethal subcutaneous challenge with 10,000 pfu of ZIKV strain FSS 13025 or MR-766 in AG129 mice, and induced a PRNT_50_ titer of 3.74 log_10_ in Balb/c mice^35^. Our results indicate that 2 doses of 0.1 µg alum-adjuvanted PIZV is sufficient to elicit a NAb titer of approximately 3 log_10_ R-mFRNT_50_ (which is similar to a PRNT_50_ titer as described in methods) and provides full protection against i.p. challenge with 10,000 pfu of ZIKV PRVABC59 in AG129 mice. While the mouse challenge studies designed and presented herein were short term and do not address the duration of immunogenicity and protective efficacy of PIZV, they provided supportive results for furthering development of this vaccine candidate. Encouragingly, reported studies utilizing a similar approach to a ZIKV vaccine have shown that a purified and formalin-inactivated ZIKV vaccine, designated as ZPIV, is immunogenic and protective in mice and non-human primates, and the protection in non-human primates lasted at least one year^31,34,45^. Additionally, data collected during a phase 1 human clinical trial with ZPIV has shown that the vaccine is well-tolerated by recipients and generates high neutralizing antibody titers against ZIKV^46^. While there are differences in the derivation and manufacturing of these two vaccine candidates (PIZV and ZPIV), both are whole-inactivated alum-adsorbed vaccine and it is therefore reasonable to anticipate comparable immunogenicity and efficacy from ongoing non-human primate and clinical studies of our PIZV candidate.

Phylogenetic studies have revealed that ZIKV exists as three genotypes (West African, East African and Asian)^21^. While lethal mouse challenge studies presented here were performed with a homotypic challenge virus, ZIKV appears to behave as one serotype^47^. Indeed, we showed that *in vitro*, pooled sera from vaccinated AG129 mice neutralized various Asian and African ZIKV strains with similar efficiency (Table 3). This finding agrees with other ZIKV vaccine studies that have investigated cross-neutralization capabilities of vaccine antisera, and suggest that a monovalent vaccine may be sufficient to protect against all three genotypes of ZIKV^33–35,43^.

NAbs play a key role in protection against flavivirus infection^30,48–52^ and have been used as an immune correlate of protection (CoP) in evaluating vaccine efficacy^53–55^. This is especially important in situations like the current ZIKV epidemic with limited and unpredictable outbreaks. Several animal models of ZIKV infection have been developed and used to define a ZIKV immunological correlate of protection by passive transfer of antibodies^31,34,35,44,46,56^. We used a lethal AG129 mouse challenge model to demonstrate the correlation of NAb levels in protection against ZIKV challenge. The type-I and type-II IFN receptors missing in AG129 mice are important for the innate antiviral response and modulating adaptive immunity^41^. Therefore, the protection in our AG129 passive transfer study can be solely attributed to the anti-ZIKV NAbs circulating in mice at the time of virus challenge. The conditions examined in the AG129 passive transfer experiment presented herein were successful in establishing a useful small animal model for testing NAb levels associated with CoP. Our results show that a NAb R-mFRNT_50_ titer of 2.2 log_10_ would provide a 70% probability of survival for ≥28 days against i.p. lethal ZIKV challenge. This level of protective NAb titer is broadly in agreement with other reported passive transfer studies for ZIKV (measured by various neutralization assays) which indicate a NAb titer of 2–3 log_10_ is protective against viremia and/or disease after challenge in mice and non-human primates^34,35,44^. While more studies are needed to fully understand the protective immune response to ZIKV, these collective data indicate that NAbs play an important role in the host defense against ZIKV infection in the model studied, can be used to establish a CoP, and are in line with what has been demonstrated for other flavivirus whole-virus inactivated vaccines^53–55^.

Collectively, our data from both CD-1 and AG129 mouse studies indicate that the PIZV candidates developed and described herein are immunogenic and provide protection against lethal ZIKV infection in mice. Importantly, when adjuvanted with alum, the low dose (0.1 µg) of PIZV was as effective as the high dose (1.0 µg) in preventing viremia and lethal infection in immunocompromised AG129 mice. This report provided pre-clinical data that was used in support of further development of the PIZV candidate, and contributed to supporting advancement to phase I clinical testing (ClinicalTrials.gov identifier NCT03343626).

## Methods

### Viruses and cells

Zika virus strain PRVABC59, isolated from a patient serum sample, was provided by the Arboviral Diseases Branch, Division of Vector-Borne Diseases, Centers for Disease Control and Prevention (ADB/DVBD/CDC), Fort Collins, Colorado. The patient was infected in Puerto Rico during the ZIKV outbreak in 2015, and the genome sequence (GenBank: KU501215.1) was generated from the specimen by the Diagnostic and Reference Laboratory/ADB/DVBD using deep sequencing. The virus isolate was made by two passages in Vero cells and one in C6/36 cells (V2/C6-1), and was confirmed free of mycoplasma and *Togavirus* cross-contamination before shipment from DVBD/CDC. Upon receiving this virus isolate at Takeda, it was amplified once (P1) in Vero cells using serum free Dulbecco’s modified minimal essential medium (DMEM) (Corning; Corning, NY). Additional ZIKV strains (DakAr41524, P6-740, MR766, and R103451) were also provided by the ADB/DVBD/CDC and amplified in Vero cells under serum-free conditions to make working stocks for this project. Vero cells were grown and maintained in DMEM containing penicillin-streptomycin (Hyclone; Logan, UT), L-glutamine (Hyclone; Logan, UT) and 10% FBS sourced from a bovine spongiform encephalopathy (BSE)-negative country (Sigma; St. Louis, MO) (DMEM-10%FBS). Recombinant trypsin (Gibco; Gaithersburg, MD) was used to maintain and dissociate cells.

### Virus titration

#### Plaque titration

Mouse viremia and virus aliquots used for mouse challenge and neutralization assays were measured by plaque titration on freshly confluent monolayers of Vero cells grown in 6-well plates as described^57^. Briefly, the first 4 mL overlay medium, containing 0.8% agarose in DMEM with 2% FBS (DMEM-2%FBS) or YELAH (0.165% lactalbumin hydrolysate, 0.033% yeast extract, Earle’s balanced salt solution, 25 mg of gentamicin sulfate and 1.0 mg of amphotericin B per liter) with 2% FBS, was added after adsorption of 100 µL/well of serially diluted samples onto Vero cells for 1.5 hrs. Following incubation for 4 days at 37 °C/5% CO_2_, 2 mL of a second agarose overlay medium containing 160 µg/mL of neutral red dye (Sigma; St. Louis, MO) was added. Plaques were analyzed on days 5 and 6.

#### 50% tissue culture infectious dose(TCID50)

To facilitate eventual large-scale vaccine manufacturing, a high-throughput TCID50 assay was established to determine the infectious titer of material used to prepare inactivated vaccine. Virus titers were determined by titration on freshly confluent monolayers of Vero cells grown in 96-well plates. At the time of the assay, frozen virus aliquots were thawed and ten-fold dilution series were made in DMEM-2%FBS, and 100 μL/well of each virus dilution was added in quadruplicate to the Vero cell plates. The plates were incubated under 5% CO_2_ for 5 days at 36 °C ± 2°C, before visual observation of the cell monolayer under a microscope for the presence of cytopathic effect (CPE) resulting from viral infection. The TCID_50_ was calculated by the Reed\Muench method^58^ to indicate the maximum dilution level of the virus that resulted in at least 50% of cell infection. The TCID_50_ titer is approximately 0.5–1 log_10_ higher than the titer measured by plaque titration described above.

### Generation and growth kinetics of ZIKV P6 sub-isolates

ZIKV PRVABC59 P1 stock, described above, was used to generate sub-isolates of the virus for producing PIZV candidates. Briefly, P1 virus was titrated on Vero cells as described above for plaque titration, and multiple large plaques were isolated and each picked agarose plug was mixed into 0.5 mL of culture medium and incubated overnight at 37 °C (P2). Three of the P2 isolates were subjected to two additional rounds of plaque purification (P3-4), and six final plaques (P4) were picked and directly amplified in individual Vero cell flasks to generate P5 virus stocks (P5a-P5f). Viral titer of the P5 stocks was determined by TCID_50_, and amplified once more in Vero cells (P6) by infecting Vero cells at 0.01 TCID_50_/cell. Two sequential virus harvests, taken three and five days pi, were pooled for each P6 culture, clarified by centrifugation, stabilized in DMEM containing a final concentration of 18% (w/v) trehalose (Pfanstiehl; Waukegan, IL) and stored at ≤−60 °C (Table 1). Growth kinetics of the P6 sub-isolates was conducted in duplicate flasks of Vero cells. Cells were infected with an MOI of 0.01 TCID_50_/cell of each sub-isolate virus in a serum free growth medium and aliquots of culture fluid were taken daily for titration by TCID_50_ assay.

### Genetic sequencing

The viral genomes of P6 sub-isolates were subjected to Sanger-based sequencing as described previously^59^. A QIAampViral RNA Mini Spin kit (Qiagen; Hilden, Germany) was used to extract viral RNA from P6 sub-isolate stocks, and 6 cDNA fragments encompassing the entire ZIKV genome for each extracted RNA sample were RT-PCR amplified using a Titan One Tube RT-PCR kit (Roche; Basel, Switzerland). After gel purification of the cDNA fragments using a Qiagen Quick Gel Extraction Kit (Qiagen; Hilden, Germany), each fragment was sequenced by automatic sequencing. Primers for RT-PCR and sequencing are available upon request.

### Purification, inactivation and formulation of vaccine candidates

A single laboratory scale preparation of the PIZV candidates (sub-isolates b and e) was generated for this discovery/pre-clinical study at Takeda. Each of the P6-b and P6-e virus stocks was amplified in Vero cells at MOI of 0.01 for 5 days in a 36 °C ± 2°C /5% CO_2_ incubator. Virus supernatants were harvested on day 3 and 5 pi, clarified by centrifugation, stabilized in DMEM containing a final concentration of 18% (w/v) trehalose and stored at ≤−60 °C. Pooled, clarified viral supernatants (200 mL each candidate) were rapidly thawed in a 37 °C water bath and treated with 20 U/mL of benzonase (Millipore; Burlington, MA) overnight at 2–8 °C to digest host cell DNA, followed by depth filtration with a 1.2 µM filter (Sartorius; Göttingen, Germany). Filtrate was applied to a Centricon Plus-70, 100 KDa MW centrifugal filter (Millipore; Burlington, MA) for a single round of concentration and buffer exchange into binding buffer (30 mL of 40 mM NaCl, 50 mM potassium glutamate, 10 mM L-Histidine and 10% trehalose, pH 7.5). The concentrated samples were diluted to 200 mL in binding buffer and subjected to Sartobind IEX nano ion-exchange chromatography (Sartorius; Göttingen, Germany). Virus samples were washed with binding buffer and eluted with elution buffer (750 mM NaCl, 50 mM potassium glutamate, 10 mM L-Histidine and 10% trehalose, pH 7.5), diluted to 300 mL in elution buffer lacking NaCl and applied to a second Sartobind IEX nano ion exchange column. Samples were eluted from the second column with buffer containing 2.7 mM KCl, 1.8 mM KH_2_PO4, 10 mM Na_2_HPO_4_, and 250 mM NaCl, pH 7.5, and applied to a Centricon Plus-70 100 KDa MW centrifugal filter for a single round of concentration and buffer exchange (40 mL) into PBS. Sample was then diluted to a final volume of 35 ml with PBS and stored at 2–8 °C.

For viral inactivation, freshly prepared 1% (v/v) formaldehyde (Fisher; Hampton, NH) was added drop-wise to each purified sample to obtain a final formaldehyde concentration of 0.02% and incubated at room temperature (~22 °C) for 14 days with frequent inversion. In a preliminary study, we had determined 7 days was required to completely inactivate virus. Therefore 14 days of formalin treatment was chosen to prepare the PIZV candidates used in this study (double the previously demonstrated duration of inactivation). Formaldehyde was neutralized with 0.04% w/v of sodium metabisulfite (Fisher; Hampton, NH) for 15 minutes at room temperature and sample was subjected to four rounds of concentration and buffer exchange (Centricon Plus-70, 100 KDa MW) with drug substance buffer (50 mL each; 10 mM NaH_2_PO_4_, 50 mM NaCl, 6% sucrose, pH 7.4). Samples were then diluted to 15 mL in drug substance buffer, sterilized through a 0.2 µm syringe filter, aliquoted into sterile stoppered glass vials, and frozen at ≤−60 °C.

Complete virus inactivation of the prepared PIZV candidates was determined by two sequential Vero cell culture amplifications of purified and inactivated sample aliquots. Sample aliquots taken on days 1, 11 and 14 were diluted (1:10 in DMEM-2%) to infect 8 replicate wells of a 96-well Vero plate. The plates were incubated and observed for CPE by TCID_50_ as described for 6 days. On day 6 pi, total supernatants from 4 replicates/sample of the 96-well plate were transferred individually into 4 wells of a 6-well confluent Vero plate for a second culture amplification and CPE observation for 8 days.

Protein concentration was determined by Bradford Protein Assay (BioRad; Hercules, CA) before each sample was formulated to 1 μg/mL or 10 μg/mL in PBS with or without dropwise addition of 0.5 mg/mL of alum (Alhydrogel, Brenntag; Essen, Germany) under aseptic conditions and incubated overnight at 2–8 °C with gentle agitation. The resulting drug product lots were then aliquoted into sterile stoppered glass vials and stored at 2–8 °C until use.

### SDS-PAGE and Immunoblotting

Samples were analyzed on 4–12% NuPage polyacrylamide gels (ThermoFisher; Franklin, MA) with a ZIKV recombinant sE protein control (from insect cells; MyBiosource,San Diego, CA). For total protein staining, gels were stained with SyproRuby (ThermoFisher; Franklin, MA) according to manufacturer protocol. For immunoblotting, protein was transferred to nitrocellulose membrane, blocked with 8% milk in wash buffer (TBS with 0.05% Tween-20) for 30 minutes, and followed by overnight 4°C incubation with a rabbit anti-ZIKV E polyclonal antibody (IBT Bioservices; Rockville, MD) diluted to 1:1000 in wash buffer. Membranes were washed, incubated with 1:1000 diluted alkaline phosphatase-conjugated goat α-rabbit antibody (KPL; Gaithersburg, MD) for 1 hour at room temperature, and then washed again prior to colorimetric development with BCIP/NBT substrate (KPL; Gaithersburg, MD) according to manufacturer protocol.

### Sandwich ELISA

96-well Immulon II HB plates (ThermoFisher; Franklin, MA) were coated with 50 µL/well of human mAb, EDE C10 or EDE C8 (Absolute Antibody; Upper Heyford, UK) diluted 1 µg/mL in coating buffer (15 mM Na_2_CO_3_, 35 mM NaHCO_3_, pH 9.6) overnight at 4 °C. Following overnight incubation, plates were blocked for 60 min at 37 °C with blocking buffer (5% milk/PBS/0.1% Tween-20). After plate washing, 10-fold serially diluted ZIKV antigen (live ZIKV PRVABC59, PIZV-e candidate, or recombinant sE) in PBS/0.05% Tween-20 was added (50 µL/well) to plates, and incubated for 60 min at 37 °C. The secondary mouse mAb, flavivirus cross-reactive 4G2 (Absolute Antibody; Upper Heyford, UK) or anti-Zika sE 1176–56 (BioFront; Tallahassee, FL), diluted to 1 µg/mL in PBS/0.05% Tween-20 was added (50 µL/well) for 60 min at 37 °C before plate washing. A goat anti-mouse IgG conjugated with alkaline phosphatase (KPL; Gaithersburg, MD), diluted to 0.67 µg/mL in blocking buffer, was added and incubated for 60 min at room temperature. After removal of the conjugated antibody and plate washing, 100 µL/well of *p*-nitrophenyl phosphate substrate (Sigma; St. Louis MO) was added at room temperature for 20 min before addition of 20 µl/well of 3 M NaOH stop solution. The optical density at 405 nm was read on a BioTek plate reader, (Winooski, VT, USA).

### Electron microscopy

Cryo transmission electron microscopy (cryoTEM) imaging was performed at NanoImaging Services, Inc (San Diego, CA USA). Briefly, samples were imaged undiluted and preserved in vitrified ice supported by holey carbon films on 400-mesh copper grids. Electron microscopy was performed using an FEI Tecnai T12 electron microscope, operating at 120 keV equipped with an FEI Eagle 4k × 4k CCD camera. Vitreous ice grids were transferred into the electron microscope using a cryostage. Images of each grid were acquired at multiple scales to assess the overall specimen distribution. The images were acquired at a nominal underfocus of −5 µm to −3 µm and electron doses of 10–25 e^−^/Å^2^.

### Mouse studies

All animal experiments were conducted in accordance with IACUC guidelines and regulations.

#### Immunogenicity study in CD-1 mice

The experiments in Swiss-ICR CD-1 mice (Charles River; Wilmington, MA) were carried out at the animal facility at Takeda (Cambridge, MA) under animal protocol 16–06–175. Six groups of 6-week old male and female CD-1 mice (n = 10/group) were immunized with 0.1 mL of vaccine or PBS placebo by the intramuscular (i.m.) route (2 × 0.05 mL injections) on days 0, 28 and 56. Blood samples were collected on days −1 (pre-immune), 27, 42 and 70 for NAb measurement.

#### Protective efficacy study in AG129 mice

The experiments in AG129 mice were carried out at the DVBD/CDC (Fort Collins, CO) under animal protocol 16–017 approved by CDC IACUC. Groups of 4-week old male and female AG129 mice (n = 5/group) were immunized with 0.1 mL of vaccine or PBS placebo by i.m. injections (2 × 0.05 mL) on day 0, and boosted once on day 28. On day 56, mice were intraperitoneally (i.p.) challenged with 10^4^ pfu of ZIKV PRVABC59. The challenge dose used was based on a prior dosing study showing mice morbidity rates (n = 5/dose group) were 0%, 40%, 60%, 100%, and 100% when i.p. challenged with 1, 10, 10^2^, 10^3^, and 10^4^ pfu of the virus, respectively. By Reed-Muench method, the LD_50_ of the virus to these adult mice was 31.6 pfu. Although both 10^3^ and 10^4^ pfu challenge resulted in 100% morbidity, the 10^4^ pfu dose resulted in a shorter average survival time (13.6 ± 1.2 days) than the 10^3^ pfu dose (18.6 ± 3.5 days). The 10^4^ pfu was chosen over 10^3^ pfu for a more stringent evaluation of vaccine efficacy.

Blood samples were collected from the tail vein on days −1, 27, 55, 58 and 79 for NAb titration or viremia analysis. Following challenge, mice were weighed and monitored daily for signs of illness for 21 days. Mice were humanely euthanized according to animal protocol upon signs of illness, including ruffled fur, breathing changes, anti-social behavior, hunched posture, twitching, paralysis, or loss of 20% original weight (whichever came first).

#### Passive immunization study in AG129 mice

Groups (n = 9/group or 6/group) of 14-week old AG129 mice were i.p. immunized with 0.1 mL of pooled day 79 sera (from protective efficacy AG129 study mice) that had been serially diluted 4-fold with PBS. Following passive immunization (16–19 hours later), blood was collected from each mouse and serum was separated by centrifugation prior to virus challenge for determination of circulating neutralizing antibody titer. Twenty-four hours following passive transfer, mice were i.p. challenged with 10^3^ pfu of ZIKV strain PRVABC59. Animals were bled 2, 4 and 6 days post challenge (N = 2–3/group/day) for analysis of post challenge viremia by plaque titration. Following challenge, mice were weighed and monitored daily for signs of illness for 28 days. Mice showing signs of illness as described in the protective efficacy study above were humanely euthanized and counted as non-survivors.

### Neutralization assays

#### Plaque reduction neutralization test (PRNT)

Mouse serum samples were tested to compare PRNT_50_ titers against various strains of ZIKVs by a PRNT assay^57^. Briefly, a target of 100–200 pfu of ZIKV DakAr41524, P6-740, PRVABC59, MR766 or FSS13025 was incubated with equal volume of 2-fold serially diluted heat-inactivated serum samples for 1 hour at 37 °C_._ Six-well plates of Vero cells were inoculated with 100 µL of the serum/virus mixtures and incubated at 37 °C/5% CO_2_ for 1.5 hours. Plates were then treated as described in the plaque titration method section. The neutralizing antibody titer was identified as the log_10_ reciprocal serum dilution that reduced virus input plaques (based on back titration of the input virus) by at least 50%. Back titration of each virus was conducted in 6 replicates of 2-fold serial dilutions of the input virus.

#### Live reporter virus microfocus reduction neutralization test (R-mFRNT)

A live reporter chimeric virus, R-WN/ZIKV, expressing prM/E of ZIKV PRVABC59 and a ZsGreen reporter protein on a WNV replication vector (containing the entire WNV genome except its prM/E genes) developed in house at DVBD/CDC laboratory was used for the R-mFRNT. The ZsGreen with a 2 A self-cleavage peptide was inserted in the chimeric WN/ZIKV^60^ at the beginning of the C gene, similar to the previously described strategy of a DENV-2 reporter construct, DENV-GFP^61^. The R-mFRNT assay with reporter virus is similar to a previously established micro-immunofocus reduction neutralization test (m-FRNT)^62,63^ but the fluorescent reporter protein expressed by the chimeric virus eliminates the requirement of immuno-staining and also allows for virus foci detection within 24 hrs pi. The integrity of the reporter gene in the reporter virus lot used was confirmed by RT-PCR, full genome sequencing, and dual-color (viral antigen and reporter) flow cytometry. The R-mFRNT assay using R-WN/ZIKV results in similar NAb titers as compared to m-FRNT and/or PRNT using wild type virus by comparison of the 2 tests with a panel of 8 human control sera samples (within 2-fold difference; ZIKV by PRNT: R-WN/ZIKV by R-mFRNT = 1.3 ± 0.3 fold).

Briefly, serum samples were diluted 10-fold in BA-1 diluent (M199 medium, 1% bovine albumin, penicillin/streptomycin, sodium bicarbonate and Tris buffered, pH 7.4) and heat inactivated in a 56 °C water bath for 30 minutes. Further two-fold dilution series of heat-inactivated samples were made in BA-1 diluent and mixed with an equal volume of R-WN/ZIKV (100–200 focus forming unit, ffu) for 1 hr at 37 °C/5% CO_2_. Each serum/virus mixture (30 µL/well) was added to a confluent Vero cell plate in triplicate for 90 min adsorption at 37 °C/5% CO_2_, followed by addition of 150 µL/well of Gibco FluoroBrite DMEM (ThermoFisher; Franklin, MA) without FBS. After incubation at 37 °C ± 2 °C/5% CO_2_ for 24–26 hours, fluorescent virus microfoci were directly live-imaged and analyzed by Celigo image cytometer (Nexcelom; Lawrence, MA). Some experiments were measured by an EliSpot reader (model ELR0681FL) (Autoimmun Diagnostika GmbH; Strassberg, Germany) after cell fixation with 4% formaldehyde for 10 min. Triplicate results were averaged, and NAb titers were expressed as the log_10_ transformed reciprocal serum dilution that reduced input virus foci (based on back titration of the input R-WN/ZIKV in the same assay) by 50% or more.

#### Reporter virus particle (RVP) neutralization assay

NAb titers from the CD-1 mouse study were analyzed by titration of serum samples with a constant amount of Zika RVPs (Integral Molecular; Philadelphia, PA) in Vero cells. Briefly, sera were heat inactivated at 56 °C for 30 min, serially diluted in assay media containing Opti-MEM (Thermo Fisher; Waltham, MA), 1% Penicillin/Streptomycin (Mediatech) and 10% FBS (HyClone; Logan, UT) and then incubated at 37 °C ± 2 °C/5% CO_2_ with RVPs (input amount empirically determined for each RVP lot) for 1 hour in a 384-well plate format. The serum/RVP mixture was then mixed with trypsinized Vero cells (4,625 cells/well) and incubated for 72 hours at 37 °C ± 2 °C/5% CO_2_. After addition of Renilla Glo luciferase substrate (Promega; Madison, WI) to each well and incubation for 15 min at room temperature, luciferase intensity was read on an EnSpire plate reader (Perkin Elmer; Waltham, MA). Data was analyzed using JMP11 non-linear 4 parameter analysis, normalized to a positive tracking control (anti-ZIKV rabbit serum) and effective dose 50% (EC50) was reported as neutralization antibody titers resulting in 50% reduction of luminescent signal intensity generated by input RVP.

### Statistical analysis

One way ANOVA was used to compare differences of NAb titers between test groups of each animal study. Correlation between circulating NAb titers and peak viremia post challenge from the passive immunization study was assessed by Spearman rank-correlation test. Comparison of geometric mean NAb titers between protected and non-protected mice was performed using a Wilcoxon rank sum test. All statistical analysis was performed using GraphPad Prism v7 (GraphPad Software; Carlsbad, CA), while R v3.3.3 software was used to perform logistic regression analysis to determine an immune correlate of protection.

## Electronic supplementary material

Suplemental Information

## Data Availability

All data generated or analyzed during this study are either included in this article or can be available upon request.
