# Supplementary material for: Purified Inactivated Zika Vaccine Candidates Afford Protection against Lethal Challenge in Mice
Source: Sci Rep. 2018 Nov 7;8:16509. doi: 10.1038/s41598-018-34735-7 (PMC6220238; doi:10.1038/s41598-018-34735-7)
Supplement: Supplementary file 1 — Suplemental Information [file 41598_2018_34735_MOESM1_ESM.pdf]

Purified Inactivated Zika Vaccine Candidates Afford Protection against Lethal Challenge in Mice

Whitney R. Baldwin[1,2†], Jill A. Livengood[1†*], Holli A. Giebler[1,2], Janae L. Stovall[2], Karen L. Boroughs[2], Stephanie Sonnberg[1], Kelly J. Bohning[1], Elizabeth A. Dietrich[2], Yee Tsuey Ong[2], Hoang K. Danh[1], Hetal K. Patel[1], Claire Y.H. Huang [2*], Hansi J. Dean[1]

[1] Takeda Vaccines Inc, Cambridge, MA, USA

[2] Arboviral Diseases Branch, Division of Vector-Borne Diseases, Centers for Disease Control and Prevention, Fort Collins, Colorado, USA

[†]Contributed equally.

[*]Correspondence and request for materials should be addressed to J.A.L. (jill.livengood@takeda.com) and C.Y-H.H. (yxh0@cdc.gov)

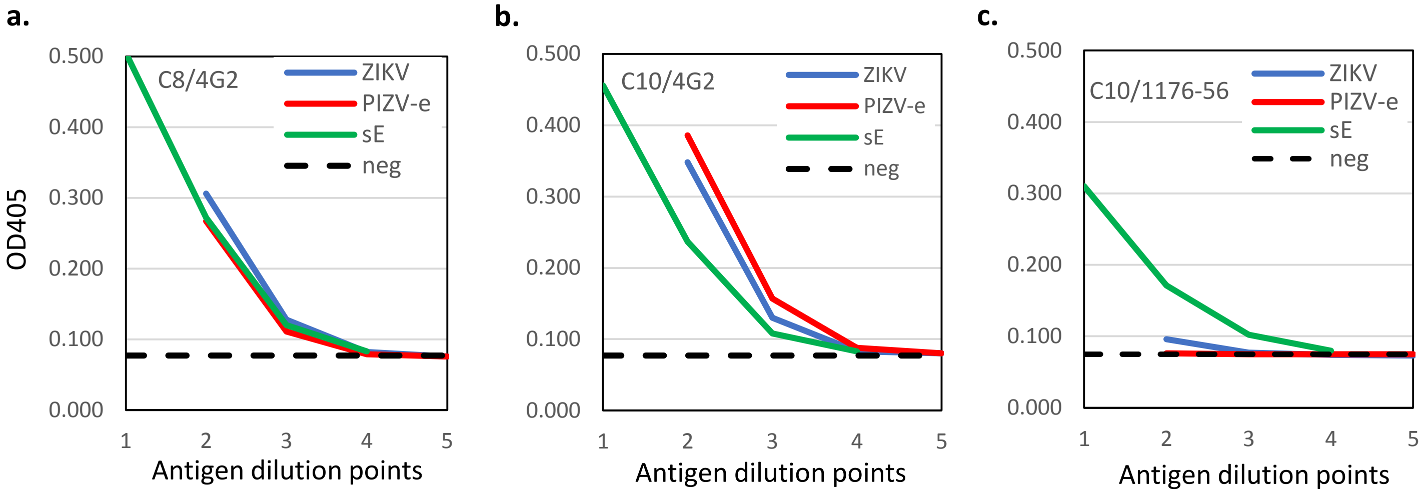

d.

Antigen input/well	Antigen dilution points (10-fold)				
	1	2	3	4	5
ZIKV ($log_{10}TCID_{50}$)	nd	6.83	5.83	4.83	3.83
PIZV-e (µg)	nd	0.60	0.060	0.0060	nd
sE (µg)	5.6	0.56	0.056	0.0056	nd

Supplementary Fig. S1a-d. Sandwich ELISA. Serially diluted antigens (10-fold) were captured by EDE C8 or EDE C10, and detected by either 4G2 or 1176-56. (a) EDE C8/4G2 (b) EDE C10/4G2 (c) EDE C10/1176-56. (d) Antigen input amount for each dilution. Positive cutoff (shaded in grey) was set at OD405 > negative control OD average (from 6 replicates) +6xSD. Shaded areas indicate an antigen input amount that resulted in a positive result for panels a and b only. For panel C, only sE was detectable by 1176-56.

a.

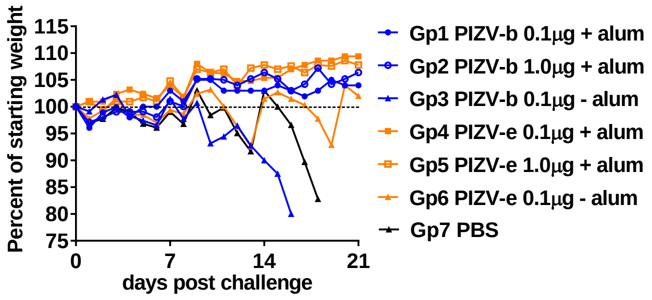

b.

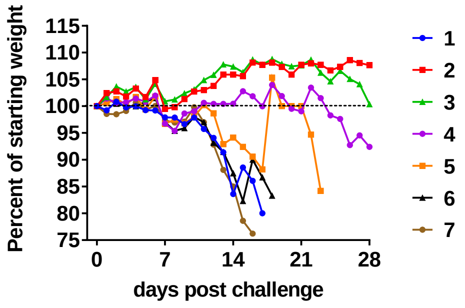

Supplementary Fig. S2a-b. Average weight of AG129 test groups post-challenge. (a) immunogenicity and efficacy study (b) passive immunization study. Average weights are represented as a percentage of starting weight.
